# α‐Synuclein aggregation landscape from phase separation to neurotoxic intermediates

**DOI:** 10.1002/1873-3468.70393

**Published:** 2026-07-01

**Authors:** Silvia Arino, Giuliana Fusco, Alfonso De Simone

**Affiliations:** ^1^ Department of Pharmacy University of Naples Federico II Italy

**Keywords:** amyloid intermediates, liquid–liquid phase separation (LLPS), neurodegeneration, protein condensates, α‐synuclein aggregation

## Abstract

The aberrant aggregation of α‐synuclein (αS) into insoluble amyloid fibrils is a hallmark of Parkinson's disease. Despite recent advances in characterising the properties of mature αS fibrils, the transient and heterogeneous intermediates that underlie cellular toxicity remain largely elusive. Here, we review the mechanistic principles of αS aggregation, focussing on liquid–liquid phase separation (LLPS) as a critical intermediate step. We discuss how the structural evolution of αS within the condensed phase governs the subsequent patterns of cellular dysfunction and pathological propagation. This framework supports an emerging state‐centric paradigm in therapeutic discovery, where the physical properties of αS condensates are modulated to mitigate the deleterious effects of its misfolding, offering a new sophisticated alternative to classical inhibition strategies.

## Graphical abstract

Alpha‐synuclein aggregation in Parkinson's disease involves a complex landscape of transient intermediates, including oligomers, fibrils and liquid–liquid phase separation (LLPS). A view is emerging in which LLPS maturation into solid‐like condensates may contribute to the formation of neurotoxic species. Understanding how these dynamic assemblies influence aggregation could open new opportunities for therapeutic intervention in synucleinopathies.

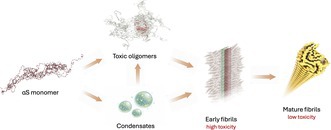

## Abbreviations

AD, Alzheimer's disease

ATP, adenosine triphosphate

ER, endoplasmic reticulum

FRAP, fluorescence recovery after photobleaching

LAG3, lymphocyte‐activation gene 3

LLPS, liquid–liquid phase separation

NAC, nonamyloid‐β component

PD, Parkinson's disease

PrPC, cellular prion protein

PTMs, post‐translational modifications

SNCA, α‐synuclein gene

SV, synaptic vesicles

αS, α‐synuclein

βS, β‐synuclein

The aberrant misfolding and aggregation of otherwise soluble protein molecules into amyloid fibrillar deposits has been associated with a number of neurodegenerative diseases, including Alzheimer's disease (AD), Parkinson's disease (PD) and systemic diseases [1]. Under these pathological conditions, protein aggregation evades quality‐control mechanisms, resulting in significant disruptions of cellular homeostasis. In the specific case of PD, these processes culminate in the deposition of intracellular inclusions, known as Lewy bodies, within dopaminergic neurons. These pathological deposits are predominantly composed of amyloid fibrils of α‐synuclein (αS), a presynaptic protein whose central role in PD was first identified by the discovery of pathological mutations in the SNCA gene, including duplication or triplication linked with early onset forms of the disease [2, 3, 4]. In PD, αS aggregates form primarily at presynaptic terminals, where their presence is associated with synaptic dysfunction and neurodegeneration of dopaminergic neurons of the *Substantia nigra pars compacta* [5]. The observation that other αS‐linked pathologies affect different brain regions, including the cortex, amygdala and brainstem, suggests that regional vulnerability is not an intrinsic property of αS alone. Rather, it reflects a susceptibility governed by the local cellular environment, the properties of the network connectivity and the robustness of protein homeostasis mechanisms.

Under normal conditions, αS is a monomeric intrinsically disordered protein primarily found in neuronal cells, where it is localised in a calcium‐dependent manner in the proximity of synaptic vesicles at the presynaptic terminals [6, 7]. It constitutes approximately 1% of total cytosolic protein component of the nervous system [8] and has been shown to possess intracellular concentration ranging from 30 to 60 μm [9]. The sequence of αS spans distinct regions with specific structural and functional roles. These regions include the N‐terminus, promoting membrane binding and spanning primarily the first 60 residues of the sequence, a central hydrophobic segment known as the nonamyloid‐β component (NAC; Residues 61–95) that is considered a key driver of aggregation, and an acidic C‐terminal sequence (Residues 96–140) modulating intermolecular interactions and calcium binding. The function of αS remains incompletely understood, although new evidence suggests a role in the trafficking of synaptic vesicles [10, 11, 12, 13, 14] as well as in synaptic plasticity [15] and learning [16]. Additional data indicate other putative functions, including the regulation of SV pools [17, 18], inhibition of the ER‐to‐Golgi vesicle trafficking [19] and interactions with mitochondria promoting the mitigation of oxidative stress [20, 21, 22, 23].

**Fig. 1:**
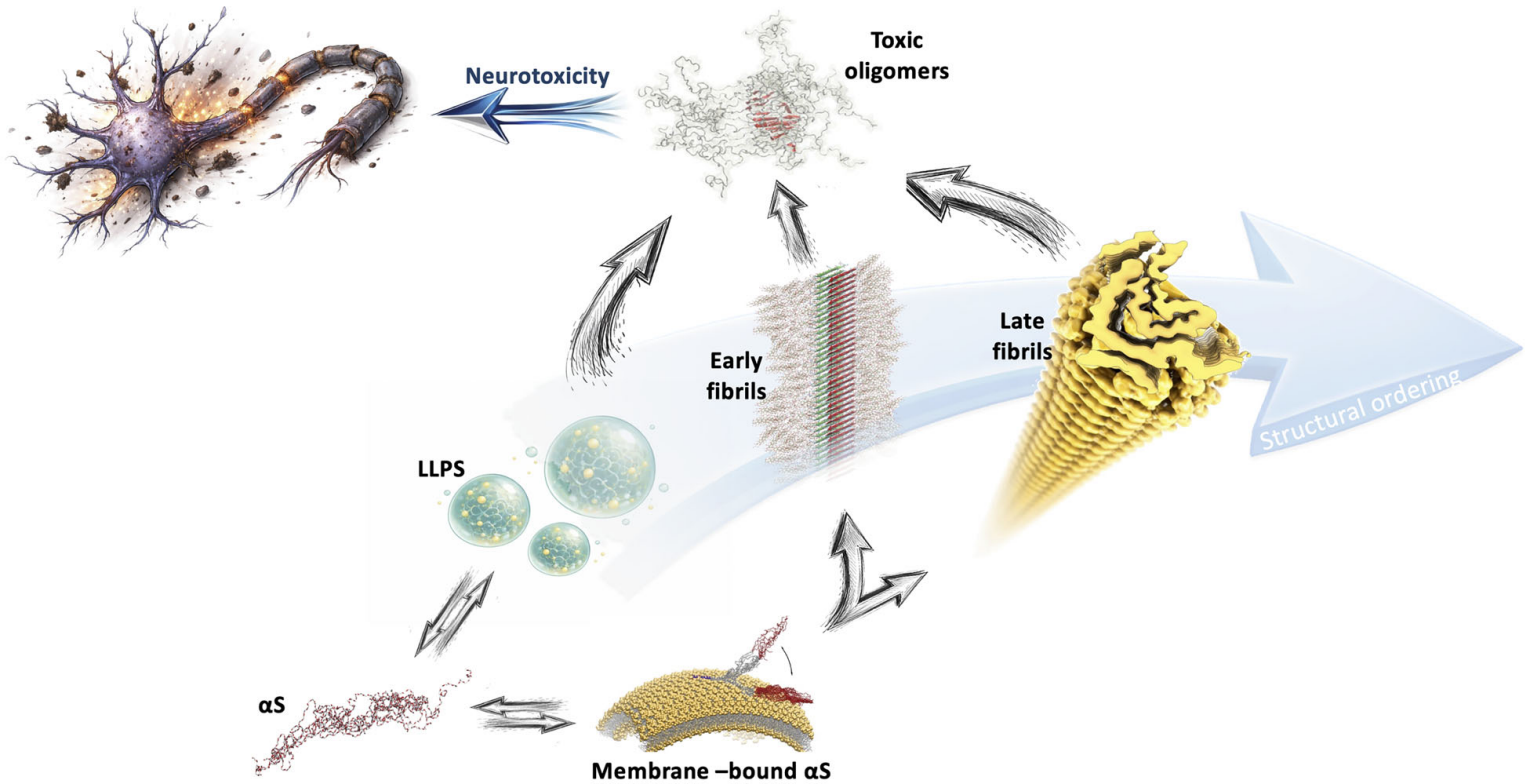
Landscape of αS aggregation. In the cytosol, αS exists as a monomeric disordered protein that is functionally in equilibrium with a membrane‐bound state enriched in α‐helical structure [27]. Under specific pathological conditions, αS can enter aggregation pathways that lead to the formation of insoluble amyloid fibrils. The aggregation landscape of αS is highly complex and can include an initial equilibrium with reversible liquid–liquid phase separation (LLPS) assemblies, which are now recognised as potent promoters of downstream aggregation into fibrillar species. Along an idealised structural ordering pathway, neurotoxic early fibrils emerge rapidly from αS condensates [28]. These protofibrillar species are stabilised by an antiparallel β‐sheet core involving short segments of the nonamyloid‐β component (NAC) region, while exposing disordered N‐ and C‐terminal regions at their surface. By contrast, late‐stage fibrils adopt a distinct topology, featuring a larger structured core organised in a parallel β‐sheet arrangement, and are generally associated with reduced cytotoxicity in neuronal cell models. Both early and late fibrillar species can also arise through interactions with cellular membranes, with their structural and toxic properties strongly dependent on membrane composition. In addition, αS can populate oligomeric states, which are widely regarded as the most pernicious aggregates in the aetiology of synucleinopathies. In this ideal pathway, αS oligomers are depicted as arising through primary nucleation within the LLPS state, fragmentation of early fibrils and secondary nucleation processes mediated by late‐stage fibrils. In this schematic representation, late fibrils of αS were adapted from reference [28], published under the CC BY‐NC‐ND 4.0 licence. The membrane‐bound conformation of αS was adapted from Fusco *et al*. [17], published under the CC BY 4.0 licence. Structural features of toxic αS oligomers were adapted with permission from Fusco *et al*. [29], Science 358, 1440–1443 (2017), © 2017 AAAS.

In the pathological context, we now possess a detailed picture of amyloid aggregates of αS, including fibrils extracted *post mortem* from patients affected by synucleinopathies [24, 25, 26]; however, many aspects of the underlying aggregation process remain unclear, including the relative contributions of primary and secondary nucleation, the role of liquid–liquid phase separation and the accumulation of oligomeric species. Mounting evidence has pointed to transient intermediate aggregates as principal mediators of neuronal dysfunction and toxicity associated with synucleinopathies (Fig. 1) [30, 31, 32, 33]. Despite their central pathological relevance, these intermediates remain the most challenging species to isolate and characterise, owing to their short lifetimes, structural heterogeneity and low abundance. The evanescent nature of these pathological intermediates has significantly hindered the development of effective therapeutic interventions for PD.

In this review, we discuss recent advances in elucidating key structural and biological aspects underlying the aggregation mechanism of αS, including emerging models that implicate liquid–liquid phase separation as a pivotal precursor in amyloid assembly. In addition to outlining how these developments are reshaping our understanding of the aggregation landscape of αS, we examine how new studies may inform novel pharmacological strategies aimed at targeting the most pernicious αS aggregates forming at the onset and development of synucleinopathies.

## Structure–toxicity relationship across the αS aggregation landscape

**Fig. 2:**
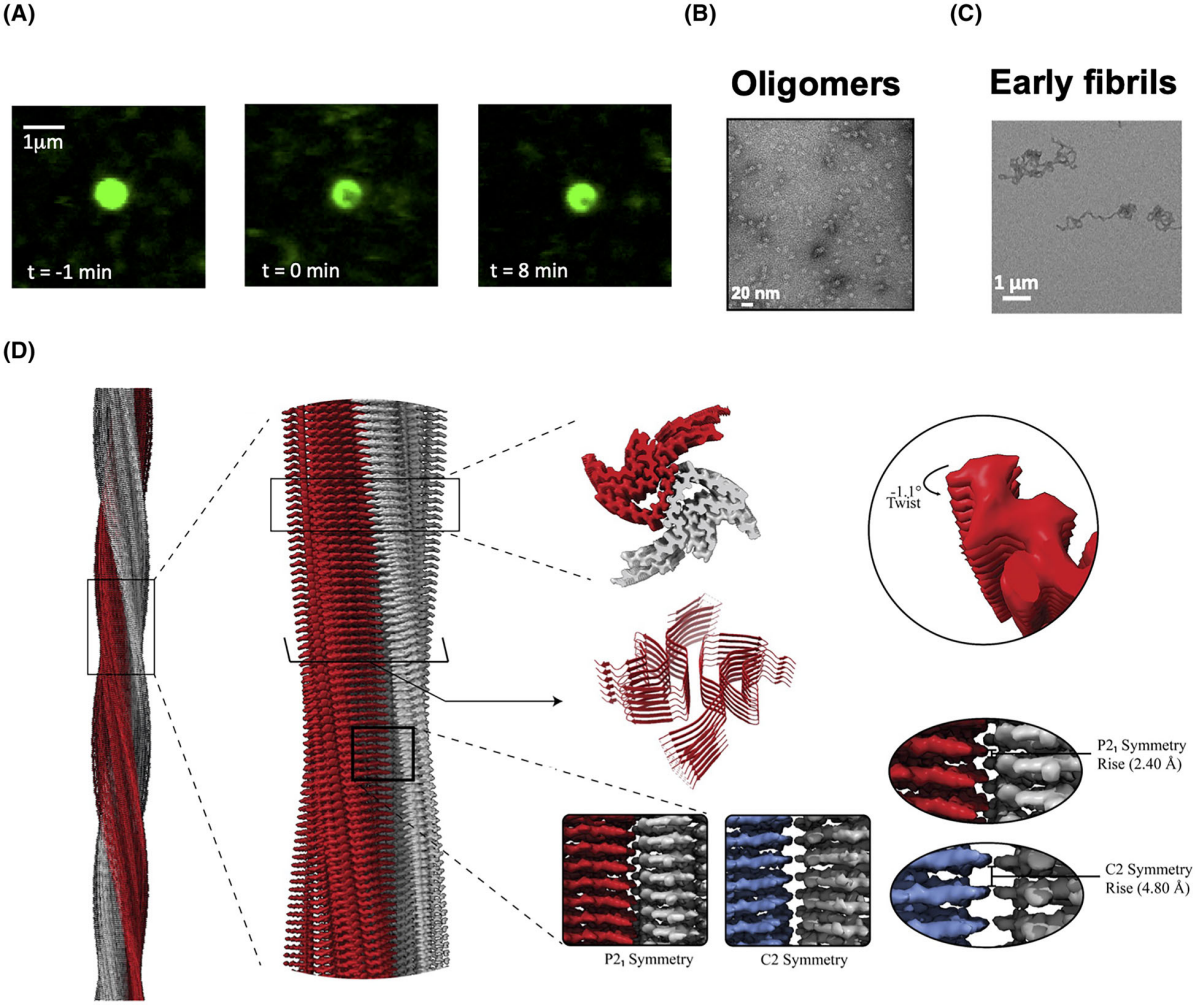
Key species along αS aggregation landscape. (A) Fluorescence recovery after photobleaching (FRAP) images of αS condensates. Fluorescence images are shown prior bleaching, immediately after bleaching (0 s) and after 8 min of recovery. Image reproduced from reference [28], published under the CC BY‐NC‐ND 4.0 licence. (B) Transmission electron microscopy of early αS toxic oligomers (scale bar, 20 nm). Image reproduced from reference [41] Copyright © 2015 National Academy of Sciences. (C) Transmission electron microscopy image of αS early fibrils emerging from LLPS after 1 day of incubation. These fibrils present a curly shape, an antiparallel amyloid‐like β‐sheet core, and disordered N‐ and C‐ terminal regions. Early fibrils have been shown to induce significant toxicity when incubated with neuronal cells (scale bar, 1 μm). Image reproduced from reference [28], published under the CC BY‐NC‐ND 4.0 licence. (D) Cryo‐electron microscopy high‐resolution structure of full‐length αS fibrils. Image reproduced from Fig. 1 in Sanchez *et al*., published under the CC BY‐NC licence [42]. This structure is composed of two protofilaments (shown in red and grey), displaying stacked β‐sheet rungs and a helical twist. Cross‐sectional views of the electron potential map reveal two α‐synuclein monomers per protofilament arranged approximately 180° from each other. Additional views illustrate twisting β‐sheet stacks, secondary‐structure organisation, axial rise measurements for P2_1_ (red) and C2 (blue) symmetries, and alternative protofilament packing modes (in‐register for C2 and out‐of‐register for P2_1_ symmetry).

Despite extensive research efforts, the underlying molecular events that drive the transition of αS from a soluble, intrinsically disordered monomer in the cytosol into aberrant aggregates, such as those found in Lewy bodies, remain debated. It is now well‐established that the aggregation behaviour of αS is sensitive to a range of physicochemical factors, including temperature [34], pH [35], salt concentration [36] and the presence of amphipathic interfaces such as lipid vesicles [37] or even the air–water interface [38]. This pronounced sensitivity has resulted in a fragmented mechanistic picture in the literature, thereby hindering the development of a unified and quantitative description of αS aggregation, including the relative roles of primary and secondary nucleation processes [35, 39, 40]. While early models described αS self‐assembly as a sequential progression from monomers to oligomers and ultimately to fibrils, new experimental evidence now supports a more complex landscape characterised by interconverting transient intermediates with distinct structural and biological properties (Fig. 1). The intermediates span a continuum of structural states ranging from liquid‐like condensates (Fig. 2A), early oligomers (Fig. 2B), early fibrils (Fig. 2C) and mature fibrils (Fig. 2D).

A major focus has been given to understanding the toxicity of intermediate species that emerge along the pathways of αS aggregation. These transient aggregates generally exhibit elevated cytotoxicity, enhanced membrane interactions and an increased capacity to disrupt cellular homeostasis. Among these species, oligomers are now widely recognised as critical toxic species [26, 38, 43], although other intermediates, such as early fibrillar assemblies, have also been shown to exhibit higher toxicity than the final mature amyloid assemblies, from which they are structurally distinct [28]. Mature fibrils may nonetheless contribute to toxicity *in vivo* through the generation of oligomeric species via secondary nucleation at their surfaces [43, 44], a mechanism that has also been described in other amyloid systems, where fibrils act as catalytic platforms for the formation of highly neurotoxic oligomers [45]. In the cellular milieu, intermediate αS aggregates preferentially accumulate to presynaptic compartments, where they perturb synaptic vesicle organisation, neurotransmitter release and calcium homeostasis [10, 14]. Such disruptions can precede overt neurodegeneration, indicating that synaptic dysfunction represents an early and functionally relevant consequence of intermediate aggregation. In addition, αS oligomers can perturb other cellular processes, one of which is the mitochondrial function [46].

Cellular quality‐control mechanisms exist to suppress the activity of toxic oligomers, which appear to be more efficiently recognised and sequestered than monomers or mature fibril [47]. αS oligomers have also been observed to exhibit extracellular activity, including engaging cell‐surface receptors, triggering synaptic dysfunction and promoting their cellular uptake [48]. Several receptors, including LAG3 [49], PrP^C^ [50] and Toll‐like receptors [51, 52], have been proposed to mediate the binding and internalisation of extracellular αS aggregates, thereby contributing to a prion‐like propagation of the pathology between neurons [53, 54], although the extent and specificity of these interactions remain unclear. In addition to receptor‐mediated effects, oligomeric αS can directly interact with biological membranes by disrupting their integrity and altering intracellular Ca^2+^ homeostasis [29, 55]. Increasing evidence, however, suggests that αS toxicity arises from pathological synergy between multiple molecular processes, including mitochondrial dysfunction and neuroinflammatory signalling in addition to membrane disruption. The convergence of these mechanisms may amplify neuronal vulnerability and contribute to disease progression. The toxicity of αS oligomers is not restricted to neuronal cells, as astrocytes and microglia have also been shown to be affected by these species, contributing to the propagation of the pathology and neuroinflammatory responses associated with PD [51].

Studies of toxic intermediate aggregates of αS spanning a range of sizes and morphologies, including oligomers and early fibrils, have identified two recurring properties that appear to underpin their shared cytotoxic activity. These include (i) a structured core formed by sequences within the NAC region and (ii) a highly lipophilic element, consistently corresponding to the N‐terminal region, which remains exposed in a largely disordered conformation at the aggregate surface. While the N‐terminal region mediates physiological membrane binding in monomeric αS, its repetitive exposure on the surface of intermediate aggregates enables cooperative and aberrantly strong membrane interactions. These interactions initiate membrane disruption through a synergic action of the structured core, ultimately leading to cellular dysfunction [29]. Consistent with this model, inhibition of N‐terminal membrane interactions, either in pre‐formed oligomers [29, 55] and early‐fibrils [28] or during aggregation *in vivo*, has been shown to markedly suppress the toxicity associated with αS aggregation. Moreover, maturation into amyloid fibrils is accompanied by structural ordering of the N‐terminal region, which consequently becomes less available to mediate membrane interactions, in line with the reduced toxicity generally associated with mature fibrillar species [28]. Beyond acting as targets of toxic aggregates, biological membranes have been shown to actively shape the formation and properties of αS intermediates [45]. Lipid surfaces can template oligomerisation by stabilising partially ordered conformations of αS and promoting local concentration effects, thereby facilitating the generation of toxic assemblies [56, 57]. The composition, curvature and charge of the membranes have been shown to strongly modulate both the structure and toxicity of αS intermediates [58, 59, 60], further reinforcing the intimate link between membrane interactions and pathological aggregation.

In addition to mediating membrane interaction, disordered surface regions, often referred to as ‘fuzzy coats’, have also emerged as key regulators of the biological activity of αS aggregates. These regions of the amyloid have been shown to possess the density and dynamics of protein condensates [61]. Moreover, Han *et al*. demonstrated that differences in the flexibility and organisation of the fibril fuzzy coats critically influence the neuronal transmission and propagation of αS fibril polymorphs, with more compact fuzzy coats correlating with enhanced seeding and spread in both neuronal models and disease contexts [62]. Disordered fuzzy coat regions are also often subjected to post‐translational modifications such as the phosphorylation of Serine 129 [63]. This and other post‐translational modifications PTMs can alter fuzzycoat dynamics, membrane affinity and aggregation pathways, thereby modulating both toxicity and propagation of αS assemblies. Together, these observations suggest that, rather than being inert moieties of the fibrils, fuzzy coats are active regions of αS amyloid assemblies that modulate both toxicity and propagation.

## αS condensates and their role in pathological aggregation

Liquid–liquid phase separation (LLPS) has emerged as a relevant step in many physiological mechanisms of the cell, including RNA metabolism, signalling and stress response [64]. Functional condensates often contain both protein and RNA, the latter regulating their formation primarily through multivalent electrostatic interactions with intrinsically disordered protein regions [65]. An increasing body of evidence, however, suggests that the phenomenon of LLPS may also play a pathological role in the initiation of aberrant protein aggregation. In particular, the maturation of these liquid‐like droplets into solid‐like assemblies has been proposed to trigger aberrant self‐assembly at the onset of neurodegenerative disorders, thereby promoting spontaneous primary nucleation, followed by rapid aggregation. This kinetic framework suggests condensation not merely as a coexisting phenomenon, but as an active promoter that reshapes the aggregation landscape by accelerating the microscopic steps leading to amyloid formation [66]. Consistent with this emerging framework, recent evidence demonstrated that the aggregation of αS is indeed preceded by liquid‐like condensates [67] (Fig. 2A), with several factors such as ionic strength, counterions, pH and PTMs influencing both the onset and kinetics of αS phase separation [68]. Much of the evidence for this transition, however, originates from *in vitro* studies performed under conditions of high protein concentration or molecular crowding. Whether αS undergoes phase separation under physiological neuronal conditions and how this process is regulated *in vivo* remain open questions.

LLPS has been associated with internal conformational rearrangements of αS, adopting a more extended state within the condensate phase [66, 69]. This process is associated with the destabilisation of long‐range transient interactions between N‐ and C‐terminal regions within the LLPS state, which in the cytosol favour a more compact hydrodynamic radius than that expected for a random coil state [68]. In analogy with mechanisms triggered in other contexts, such as calcium bursts [70], the disruption of the N‐to‐C terminal interaction is coupled with conformational rearrangements within the NAC region, thereby reducing the energetic barrier for the nucleation of aggregation‐competent species [69]. Taken together, these observations provide a compelling mechanistic rationale for the enhanced aggregation observed within αS condensates [71, 72].

The biological relevance of the evolution of αS condensates into solid‐like states becomes most evident when considering the properties of the fibrillar species emerging from these assemblies. The intermediate species of αS that appear during the early stages of condensation possess structural properties that are fundamentally distinct from those emerging as the assemblies age. In particular, under salting‐out conditions, early fibrils emerging from αS condensates exhibit a curly morphology and possess an antiparallel β‐sheet core composed of a short segment of the NAC sequence and disordered regions at both the N and C termini [28]. These protofibrils induce pronounced cytotoxicity when incubated with neuronal cells, including mitochondrial dysfunction and oxidative stress. By contrast, late‐stage fibrils that arise as the condensed phase ages exhibit markedly reduced toxicity, a straight and elongated morphology, and a larger structured core based on a parallel β‐sheet topology [28].

αS condensation is not strictly confined to pathological aggregation but has also been observed in physiological contexts. For example, transient electrostatically driven interactions between the juxtamembrane domain of VAMP2 and the C‐terminal region of αS were shown to promote phase separation *in vitro* [73], with the resulting condensates selectively sequestering synaptic vesicles and presynaptic proteins such as VAMP2 itself and the SNARE regulators complexin‐1 and complexin‐2. Further studies have clarified that αS condensation is not exclusively driven by self‐association, as co‐condensates with the proinflammatory amyloidogenic protein S100A9 were shown to form under crowding conditions [74]. These heterotypic assemblies fundamentally reshape the aggregation behaviour of αS, both by modulating nucleation behaviour and by biasing the selection of specific fibrillar polymorphs, thereby providing a direct link between inflammatory microenvironments and altered aggregation outcomes. αS condensation also involves some endogenous regulators including β‐synuclein (βS), which acts as a negative regulator of αS phase separation by reducing molecular mobility within co‐condensates. Indeed, βS was shown to suppress the fusion, growth and maturation of αS condensates, although it appears incapable of reversing the transition once the process of condensation has been initiated [75]. The evidence that mutations of βS associated with dementia with Lewy bodies impair its ability to inhibit αS condensate coalescence and αS‐induced dopaminergic neurodegeneration in *Caenorhabditis elegans* indicates that the αS LLPS can be finely regulated *in vivo* to avoid aberrant aggregation.

Taken together, these data support an emerging view in which αS phase separation is a tunable precursor to aggregation, promoted by conformational reorganisation and maturation of the condensate. The fine balance between physiological homeostasis and aberrant aggregation is thus dictated by the interplay of environmental conditions, molecular partners and endogenous modulators.

## New opportunities for therapeutic targeting of αS condensation

It is becoming increasingly evident that without a rigorous understanding of the physiological role of αS, the rational development of molecular therapeutics to prevent its pathological aggregation remains a complex challenge [76, 77]. In this context, the recent recognition of αS condensation offers new opportunities for pharmacological intervention aiming at modulating the transition towards pathological aggregation while specifically stabilising the protein in its physiological state. A view is therefore emerging in which effective compounds are not expected to function as classical high‐affinity inhibitors, but rather as molecular remodellers of the underlying aggregation landscape. In particular, such agents would bias the system towards aggregation‐resistant states, for example by impeding the maturation of the condensed phase into fibrillar species [78]. In this context, a growing body of evidence indicates that chemically diverse compounds (Table 1), including small molecules, designed peptides and endogenous polycations, can modulate αS behaviour by altering LLPS properties and downstream aggregation pathways. Collectively, these observations have reframed αS pharmacology from a binding‐centric perspective to a state‐centric view, in which ligands influence toxicity by controlling the physical and dynamical properties of protein assemblies.

**Table 1:** Small molecules interplaying with αS condensation.

Molecule	Chemical properties	Modes of action	Preclinical results
Spermine	Endogenous polyamine	Promotes condensation through electrostatic screening and modulates condensate dynamics [79]	*In vitro* studies
Claramine	Amphipathic polyamine derivative	Stabilises liquid‐like αS condensates [80]	*In vitro* studies
Cyclic macropeptides	Synthetic cyclic peptides	Bind the αS C‐terminal region and modulate intermolecular interactions involved in condensation and aggregation. [81]	*In vitro* studies
ATP	Cellular nucleotide	Acts as a biological hydrotrope modulating intermolecular interactions within protein condensates [82, 83]	Demonstrated for other protein condensates

Small endogenous metabolites have also been found to influence biomolecular condensation (Table 1). In particular, ATP has been shown to act as a biological hydrotrope capable of modulating phase separation behaviour in different protein systems by weakening intermolecular interactions within the dense phase [82, 83]. Endogenous polycations such as spermine were shown to act as noncanonical molecular glues that promote the condensation of both αS and Tau while enhancing the internal dynamics of the droplets and simultaneously suppressing fibrillisation [79]. These effects are attributable to charge neutralisation and stabilisation of highly dynamic condensates that remain accessible to cellular clearance pathways, including autophagy. The polyamine claramine was also shown to selectively target and stabilise the condensate state of αS, thereby preventing the formation of β‐sheet–rich aggregates, reducing internal droplet rigidification and inhibiting both primary and secondary nucleation processes [80]. The inhibition of liquid‐to‐solid transition in αS condensates was also attributed to phenolic compounds derived from traditional Chinese medicinal formulations [84] as well as to *de novo*‐designed cyclic macropeptides [81]. These peptides enable a high degree of control over the kinetics of droplet ageing, through a concerted interplay of electrostatic and hydrophobic interactions, combined with compact architectures and strategically positioned aromatic residues in the sequence. These molecules were shown to interact with the C‐terminal region of αS, promoting chain expansion and reweighting interdomain interactions that favour LLPS, thereby emphasising the possibility of regulating the condensate phase using tailored molecular scaffolds.

Taken together, studies of small‐molecule modulators of αS condensates have rapidly generated a robust framework to outline preliminary principles to guide future therapeutic design. In particular, polar and flexible molecules tend to enhance internal droplet dynamics, whereas rigid polycationic or planar aromatic compounds promote condensate stiffening. These observations therefore provide a mechanistic basis for the rational modulation of αS phase behaviour [68, 78].

## Conclusions

Over the past decade, a growing body of evidence has established that αS aggregation unfolds across a complex and dynamic landscape, with LLPS now recognised as a central and previously unappreciated component of this process. These advances highlight that neuronal dysfunction is driven not by a single aggregate species but by a continuum of structurally and dynamically distinct states whose properties are finely tuned by environmental conditions, interacting partners and cellular regulatory mechanisms. LLPS is therefore emerging as a central triggering element within this continuum, integrating concentration effects, kinetic acceleration and structural selection to generate pathogenic intermediates. This new framework reshapes our understanding of how aggregation initiates, propagates and exerts toxicity in synucleinopathies.

A deeper mechanistic understanding of how such dynamic processes gives rise to toxic intermediates *in vivo* remains crucial, both for establishing a quantitative knowledge of αS aggregation and for enabling the rational design of therapeutic strategies aimed at intervening at the most pathogenic stages of the aggregation cascade. Indeed, this emerging data open new conceptual and practical avenues for therapeutic intervention. Rather than targeting αS through classical high‐affinity binding, a state‐centric strategy that modulates condensate properties, aggregation kinetics and the maturation of pathological assemblies offers a promising new alternative. Future efforts integrating high‐resolution structural approaches, quantitative kinetics and cellular models will be critical to define the detailed molecular determinants governing αS phase behaviour and toxicity. Such integrative understanding will be critical for translating these insights into rational strategies aimed at restoring proteostasis and preventing neurodegeneration in PD and related disorders.

## Author contributions

SA, GF and AD were involved in the conceptualisation. SA conducted the literature search. AD and SA designed the figures. All authors reviewed, edited and approved the final version of the manuscript.
